# Classroom Response System in a Super-Blended Learning and Teaching Model: Individual or Team-Based Learning?

**DOI:** 10.3390/pharmacy8040197

**Published:** 2020-10-24

**Authors:** Maryam Malekigorji, Taher Hatahet

**Affiliations:** School of Pharmacy, Queen’s University Belfast, Belfast BT9 7BL, UK

**Keywords:** classroom response system, flipped classroom, team-based learning, blended teaching and learning, interactive teaching, TurningPoint, student engagement

## Abstract

Building an interactive environment during learning experience is sometimes hindered by student numbers in class, their sociocultural differences and limited teaching time, which may reduce student engagement. In this study we provided a super blended teaching and learning model by hybridising Classroom Response System (CRS) with Flipped Classroom (FC) and Team-Based Learning (TBL). CRS allowed learners to use their smart devices (e.g., phones, tablets and laptops) to respond to a variety of numerical, multiple-choice, short-answer and open ended questions posed during live classes and encouraged them to engage with classroom activities. Our Flipped-CRS (F-CRS) approach required the students to preview the e-learning material and watch the recorded lectures before the sessions and apply their knowledge within the session, either individually or as teams, by answering questions using TurningPoint CRS software. Learners provided positive feedback regarding F-CRS and the application of super blended teaching and learning model demonstrated a substantial increase in student collaboration and enhanced their motivation, engagement, attendance and academic performance, especially while using F-CRS approach in teams. Our super blended approach enabled educators to monitor student engagement throughout the year, facilitated formative assessment and assisted teachers to create crude class performance prediction in summative assessments.

## 1. Introduction

Nowadays, education is focusing on providing students what they need to succeed more than ever in the digital networked world of the 21st century, where the focus on student behavior, learning, engagement, performance and interaction becomes the heart of the learning process [1]. Therefore, it is essential for Higher Education to embed creativity and innovation in the curriculum design at a higher extent that enables students to actively participate in the process of knowledge construction through communications [2,3,4]. In line with other disciplines, pharmacy education has been moving towards student-centered learning to transform learner from being an observer to an actor in the learning process [5].

Active learning is well documented in the literature, in which learners mentally and/or physically interact and engage in learning activities that forces them to reflect upon ideas and is known to bring positive outcomes with regards to student learning experience and academic performance [6,7]. Integrated active-learning strategies in the curriculum are part of the accreditation standards for Master of Pharmacy (MPharm) and Doctor of Pharmacy (PharmD) programs in the UK [8] and the United States [9], respectively.

Changing demands in education marketplace, student work habits and increased desire to design flexible education in Higher Education have motivated educators to implement different active learning approaches including role playing [10], problem solving [11], Team-Based Learning (TBL) [12,13] and Flipped Classroom (FC) [14,15], in order to allow learners to develop important and updated professional competencies. Digital communication, media, and computing technologies with fast and easy access to information, which allow learners to interact, collaborate and share skills and experience in a more open world, have radically changed the teaching paradigm and the conventional education system. Nowadays, educational technologies offer interactive learning environment that provides opportunities for students to track their own progress over the course and assist learners to improve their engagement and performance [16].

Different cognitive motivational factors such as self-efficacy and goals achievement influence active learning in which students’ interpretations of successes and failures affect subsequent self-efficacy beliefs, their engagement, and interactions [17]. It is also well perceived that motivation affects peer interactions and learning specially while working as teams. Motivation and perceptions of teammate contributions are influenced by team enjoyment that attracts logy of educational matters to students. This is with the implication that learners who perceive that the team interactions and engagement are adding value to their education will enjoy learning and appreciate learning outcomes at a higher extent [18].

### 1.1. Classroom Response System

Different digital learning and assessment technologies are now brought up to reinforce active learning methodologies such as in class polling via CRS including TurningPoint polling software [16]. Poor in-class engagement with large number of students is very common and almost inevitable in a conventional teaching model as learners have little or no opportunity to interact with the instructor. This imposes challenges for students to participate in activities and provide feedback with their opinions, knowledge, or progress. To overcome this problem, CRS (named also as Audience Response System (ARS) or Personal Response System (PRS)) has emerged as a popular active learning tool, which provides equal opportunities to all leaners to take part in class activities and captures student feedback immediately [19]. CRS have been applied in medical education in a variety of health profession courses and disciplines including pharmacy, medicine, dentistry, and nursing since the 1970s, but have become more popular in the last 15–20 years [20,21]. This learning and teaching technology enables learners to interact with educator and peers in an anonymous (or identified modes) and engaging way by answering questions or participating in discussion via clickers or smart devices.

However, although the use of CRS is highly desirable and have been applied significantly over the last decade, it should not be crammed into already heavy class time and can only reinforce the learning outcomes if it well integrated into the course. Moreover, factors such as the type and design of activities and the quality and the variety of the questions significantly influence on the degree of learner’s interaction and engagement [22]. Until recently, CRS suffered from technological limitations. Traditional CRS enabled learners to select correct answers to only multiple-choice questions by using electronic hardware (clickers) and allowed educators to rapidly collect and analyse student in-class responses [23]. However, currently CRS enables respondents to use smart devices such as smartphones, tablets, and laptops as cost efficient alternatives to hard clickers to answer a variety form of questions.

CRS now provides the opportunity to generate points for discussion in the class, inform the direction of teaching, apply games and surveys, and implement free form answers. The frequency of responses can be shown in the class in order to provide formative (and, in some cases, summative) and timely feedback, enhance motivation and engagement and facilitate class discussion around the question topic in large classes [22]. Anonymously answered questions with instant feedback as cognitive intercession strategies and systematic desensitisation techniques applied in CRS can be an ideal form of assessment as anonymous answering reduces anxiety levels in students unlike other forms of response such as raising hands in active-learning situations [24]. Analysing a sample of over 10,000 pharmacy students showed that at least 1 in 5 scored high for communication apprehension [25], which is a phrase used for “an anxiety syndrome associated with either real or anticipated communication with another person or persons” [26]. Therefore, CRS may be of particular relevance in pharmacy and pharmacy related education.

It is well documented that CRS can improve student cognition, data retention and academic performance by encouraging engagement with course content, educators, and peers as well as improving in-class attention and concentration, since in most cases learners are expected to be prepared and answer live questions [27,28,29]. Moreover, engaged students make conscious decisions to apply their knowledge, skills, and resources to improve their understanding and learning [26]. Wieman and Perkins stated that embedded CRS courses can have significant positive impact on students educational experience as it offers learners fewer structured concepts, assists them to identify the key points and their interrelationships, and subsequently recasts the main learning outcomes into a form in which they can appreciate [30]. Therefore, CRS can be also an ideal approach to deliver revision sessions where students work together to answer questions. CRS immediate student feedback system also benefits the instructors to sample learners’ opinion, examine their understanding and learning to adjust the course structure and the teaching mode [29]. The most significant challenges for student include adjusting to a new learning style and remaining focused when multiple perspectives are discussed. Main challenges for instructors while applying CRS are the time and skills required setting up CRS session, creating effective questions, ensuring adequate coverage of course material, dealing with student negative reactions who are being monitored and responding to instantaneous student feedback [31]. Therefore, more systematic and detailed investigation is required in a broader range of CRS context.

### 1.2. Flipped Classroom

Flipped classroom (FC), as another example of a technology-supported pedagogical approach, which has become popular in recent years, enables educators to provide less critical and low cognitive materials to learners to be previewed outside the classroom, which frees up class time to be used to spend more time on higher level learning, critical thinking exercises and problem solving practices [32]. In-class instructions and specially developed narrated lecture material online are normally provided to students in advance of class to allow them to prepare themselves individually or in groups. This enables the learner to receive the most benefit from time spent in live classes that foster their engagement via different collaborative activities such as providing feedback, solving problems, introducing advanced concepts and discussions, which ultimately transfers the responsibility and ownership of learning from instructor to learner [33]. In FC approach, multimedia content such as recorded lectures is often seen as the key mean of accessing knowledge for learners. This provides a degree of flexibility for students to skip subjects they understand well and pause or replay contents they find more challenging for revision, which is beneficial especially for those studying in a foreign language [32].

Pierce and Fox investigated the effect of FC model in a pharmacotherapy module and reported that students appreciated the flipped approach and its pedagogy as a preferred mode of learning in comparison to the traditional lecture-based model, which significantly enhanced their application of knowledge, critical thinking and problem-solving skills through formative assessments and participations [33]. These are critical skills for pharmacy and pharmaceutical disciplines, which enable students acquire competency and capacity for their future career [34]. This is also in line with a recent study, which suggests that FC approach enhances active learning for MPharm students on medication distribution systems [35]. However, some reports reflect that pharmacy students may lack comfort with technology-driven teaching and learning approaches related to the accessibility and difficulty with the directions and therefor FC instructor should arrange additional introductory sessions to train students step-by-step [36]. Moreover, although FC showed substantial benefits in pharmacy education [34], some learners still do not consider it as helpful as in-class demonstrations, which might be related to their past educational experience [35] and therefore its application in pharmacy education is yet to be determined.

### 1.3. Team-Based Learning

Team-based learning (TBL), as a notable active-learning strategy implemented in healthcare education, is a form of cooperative learning model that focuses on critical thinking, communication, student accountability, and engagement in learning [37]. TBL was first introduced in the DPharm program in 2008, when a traditionally lecture-based endocrine module was replaced by a TBL module, in which learners dealt with solving patient cases, which revealed a reduction in face to face classroom time by 40% without posing a negative impact on student outcomes [38]. Later, TBL was introduced into pathophysiology and therapeutics courses in pharmacy education in order to improve learners’ problem-solving skills while integrating science to practice. Results show that 20% higher team score was achieved in comparison with individual performance on assessment indicating the benefit of peer-teaching. Moreover, student satisfaction and their understanding of course material were significantly improved [39]. Other recent reports of successful implementation of the TBL approach in medical [40], pharmacy [41] and nursing [42] curricula as a small component, for an entire module, or an entire course are examples showing the importance of TBL approach in enhancing students learning experience, their academic performance and competency in their future career.

The TBL approach requires students to acquire foundational knowledge prior to class by advanced preparation, including reviewing literature and/or instructor-prepared handouts, and completing clear instructor-developed unit objectives. By active classroom participation and student discussion in small groups, TBL creates an environment that enables students to develop and improve their learning and to apply, analyse and evaluate knowledge in order to complete tasks that are significant and meaningful and assist them to understand real-world problems [12,13,43]. Moreover, facilitated small group discussion in the TBL interactive environment helps learners to identify gaps in their understanding, which subsequently urge them to self-directed study and research [43].

However, embedded TBL courses need significant adjustment and changes in teaching and learning process by both educators and learners. Successful implementation of TBL requires careful team allocation and management which considers team permanence, diversity of resources, team accountability and the ability to communicate clearly. Learners in high-functioning teams require to develop trust and clear team expectations as groups of divergent learners. Diversity in teammates’ educational and cultural background and academic and personal experience assist teams to achieve higher degree of communication, engagement, and performance [44].

It is crucial that teams and individual learners in TBL approach receive frequent, constructive, and timely feedback from their instructor. The feedback can be provided short after the readiness assessment test for individuals (iRAT) and teams (tRAT) at the beginning of the sessions, which can be followed by group discussion in order to enhance inter-team interactions. Therefore, TBL as a student-centered approach assists educators to identify gaps in understanding and examines learner knowledge with follow-up questions [45].

One of the challenges in designing TBL exercises is to create activities in which student interaction is encouraged via the application of collective knowledge, fundamental course concepts, skills, and values to find a specific solution or to make and defend a decision. Challenges for students in adjusting to the TBL format generally is around the team dynamics, allocating responsibilities fairly and effectively and building trust. This might be due to the fact that team collaborations instead of competing individually during learning process might be completely a new experience for some learners [45]. Learners with extensive passive education background may also find it challenging to switch to the TBL model and break the learning cycle of cramming information for the completion of assessments. Moreover, maintaining a balanced and realistic relation between the volume of assigned pre-class tasks and the amount of time learners have to prepare themselves prior to the TBL session can be sometimes challenging. Providing information regarding the pre-session assignments and supplementary material well in advance, i.e., at the beginning of the course, allows learners enough time to engage with the material ahead of classes effectively, especially for those who are new to the TBL approach.

### 1.4. Blended Learning

Blended learning (BL), sometimes referred to as hybrid or inverted learning, has attracted great attention as one of the most effective and popular mode of learning since 2000 [46]. Although there are still variations and ambiguity in defining BL, all definitions address this approach as a strategy to combine two or more than two instructional modalities and methods or online and face-to-face instruction [46]. BL was one of the short-term forces driving technology adoption in Higher Education, which considers the characteristics of digital technology and information communication technologies that provides flexible, timely and continuous learning to students with different learning capabilities. While BL approaches have raised some concerns over the years such as lack of student access to educational technologies, challenges in embedding a BL treatment effect into an existing module, course or curriculum, and demanding great extent of student self-regulation; they have shown excellent results in enhancing learner’s motivation, interactions, engagement and subsequently have improved academic performance if they are designed and applied efficiently [47,48]. BL, as an effective approach for accommodating an increasingly diverse student population in higher education, have also shown to improve course satisfaction [49,50,51] as well as enhancing students’ sense of community [52] in comparison with lecture-based learning.

### 1.5. Study Rationale

In previous studies, we investigated the effect of team and its dynamic in a BL model on students’ perception, engagement and performance while FC and TBL learning were hybridised with face-to face learning (FTBL) in undergraduate pharmaceutical science/biotechnology courses. The application of FTBL approach resulted in higher course satisfaction and academic performance and enhanced student communication and problem-solving skills [41,43]. We aimed to allocate more preparation time prior our engaged sessions and actively train students to create their own learning path with respect to their future orientations which has shown to contribute substantially to the enhancement of student’s competitiveness and employability [53]. As a result, both FC and TBL approaches are key component in our current educational study.

In this article we assess the effect of concurrent implementation of CRS and FC models for individual Level 1 BSc classroom activities in IF-CRS blended approach as well as hybridising CRS, FC and TBL models in Level 2 BSc super-blended TF-CRS model at Queen’s University Belfast (QUB) joint College in China (CQC).

## 2. Methods

### 2.1. Blended Learning Models

Level 1 (L1) and level 2 (L2) students studying BSc degree in Pharmaceutical Science/Biotechnology at CQC were exposed to BL models, in which FC, TBL and CRS were hybridised with face-to-face and lecture-based learning across several modules. Learners were provided with pre-session recorded lectures and supplementary directed reading materials while assigned pre-class tasks prior attending active classes either individually (L1 in IF-CRS model) or in teams (L2 in TF-CRS model). Two modules in each level (organic chemistry and physical pharmaceutics in L1 and industrial pharmaceutics and medicinal substances in L2) were selected to embed IF-CRS and TF-CRS learning models for the entire academic year, which was introduced at the beginning of the term.

**Figure 1:**
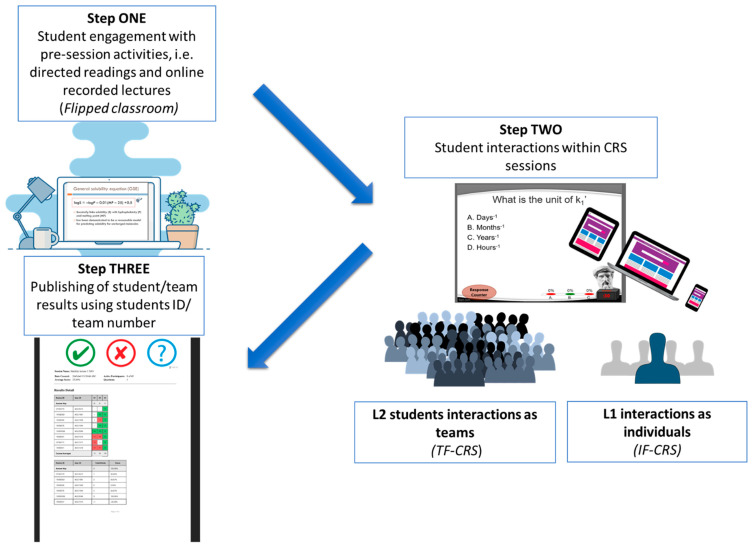
Schematic representation of the chronological order of events in IF-CRS and TF-CRS approaches during one academic year.

Teams in L2 were asked to sit in different locations of the classroom in each session in order to provide an equal chance for all learners to experience how the location of their seat may affect their learning. Students were encouraged to apply their knowledge by answering a full range of interactive question types during the class via the application of TurningPoint CRS technology (Turning Technologies (TP)), which also enabled the educators to track attendance. TP, as an interactive polling software, facilitates both web-based environment and student response mobile application to engage, which assisted our learners to use any smart device to view, interact and respond to activities in real time either anonymously or by name (if asked). Where team interaction was required (L2), the team leader, assigned by members, collected the general team view, and submitted agreed responses. Meanwhile the instructors were able to message individuals or all students at any point during the sessions. TP polling questions were launched within the PowerPoint presentation in slideshow mode with appropriate limited time allocated to respond. The results in a form of frequency of individual or team answers were shown on screen after closing the poll for each question. Following each session, individual or team scores were published to provide on-going feedback as a form of formative assessment and to assist students to track their answers and progress. Students were notified that individual (L1) or team (L2) prizes would be awarded for the first, second and third high scores at the end of the course. It was anticipated that previewing class content and completing activities prior to the flipped classes will assist students to become active learners while their performance can be monitored and analysed by instructors as an indication of understanding, the degree of interaction, engagement and learning progress to justify teaching. After each session, student/team scores were recorded using their student number or team number and archived. Figure 1 shows a schematic representation of the chronological order of events in our blended/super-blended teaching and learning approaches during one academic year. The sequence was repeatedly used throughout the entire academic year. Both levels were invited to CRS sessions twice a week and final examination was applied at the end of the academic year.

### 2.2. Creation of the Questionnaires

Following piloting, and receipt of QUB ethical approval (003PMY2019), two sets of similar questioners were introduced to investigate BSc L1 and L2 students’ perception regarding IF-CRS and TF-CRS approaches, respectively. Data were collected by means of online questionnaire (SurveyGizmo), which has been deemed the most suitable approach, as it removes factors related to other methods, such as paper-based questionnaires, which may limit response rates, as well as negating the need for students to be on-site in order to respond, and thus increasing convenience [54]. The questionnaires were developed with reference to our previous studies [41,43], existing literatures [55,56], and feedback derived from discussions with faculty staff and student representatives. The resultant questionnaire made use of Likert-type attitudinal, rating, and ranking questions, in addition to open questions, allowing for categorical data to be captured and additional detail and discussion to be obtained. The questionnaire itself was divided into four sections:

- Section A (two questions) that involves open-response questions, which consider the likes and dislikes of the blended/super-blended teaching models, to gather qualitative information about perceived issues which may be technical, cultural, etc. in origin.
- Section B (12 questions), which examines the student’s views on FC coupled with CRS (F-CRS) and associated skills development, gauging their opinions on the usefulness of F-CRS as an approach, and the ability of this technique to improve their academic performance.
- Section C (17 questions) that investigates students’ general views on blended sessions
- Section D (four questions), which relates to demographic information but does not include the collection of any identifiable information.

A cover sheet was prepared for the questionnaires to: (a) outline the purpose of the research, (b) provide a definition of “FC”, “TBL”, “IF-CRS” and “TF-CRS”, (c) give a predicted time required to complete the questionnaire (20 min, piloted by postgraduate students), and (d) provide assurance that the participation is voluntary and that the data (which is non-identifiable) will only be used for research purposes. In the aim of maximising response rates, the questionnaire was relatively short, and the questions were largely in a closed-question format [56].

Collected data were processed using IBM SPSS, USA (Version 22) software, and analysed using appropriate statistical tests with *p* < 0.05 set a priori.

## 3. Results

### 3.1. Demographic Data of Participants

Table 1 presents the demographic information of the students that participated in the survey. More females participated in the study than males as the majority of the student cohort registered on the course were female (65 out of 104 and 61 out of 74 in L1 and L2, respectively).

### 3.2. Students’ Perception on IF-CRS and TF-CRS Learning Approaches

Table 2 indicates student perception on performance, assessment, and the suitability of IF-CRS/TF-CRS in comparison to traditional teaching methods extracted from online questionnaires. In general, both L1 and L2 students perceived IF-CRS and TF-CRS learning approaches positively, respectively. The lowest mode observed was at 4, equivalent to agree in the Likert scale. Although TF-CRS was ranked higher than IF-CRS in general, the difference was not found to be statistically significant (*p* > 0.05).

**Figure 2:**
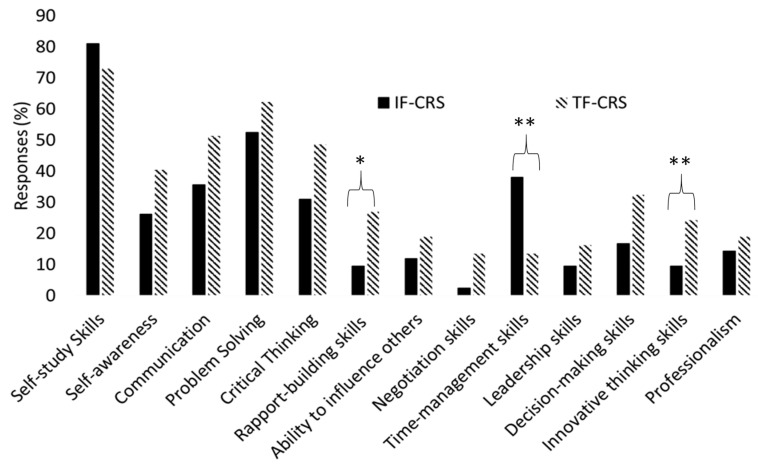
Student’s perception on skills improved and/or developed during IF-CRS (*n* = 74) and TF-CRS (*n* = 104) learning approaches. * denotes *p* < 0.01; ** denotes *p* < 0.005.

Figure 2 shows developed or improved skills in L1 and L2 study groups regarding IF-CRS and TF-CRS approaches in percentage, respectively. The ‘developed or improved skills’ only describe the skills as perceived by the students and were not investigated by the educators. Self-study was the highest reported skill among both groups. In general, skills were ranked by L2 students significantly higher than individual learners in L1 (*p* < 0.05). However, L1 group ranking surpassed L2 students in self-study and time-management skills, which are normally considered as the main transition challenges for first-year students [57]. Moreover, individual learners received negligible benefit (<10%) from IF-CRS approach to develop or improve negotiation, rapport-building, leadership, and innovative thinking skills which most likely to be achieved by teamwork.

Table 3 represents examples of student positive and negative feedback toward IF-CRS and TF-CRS approaches and associated learning activities. Both L1 and L2 groups appreciated the impact of the learning mode on course understanding, which required them to preview before engaging in interactive CRS sessions. Students found the full range of interactive questions and practices during the sessions beneficial for their exam preparation and revision. Moreover, TF-CRS was greatly appreciated in terms of improving communication and teamwork skills. In addition, L2 leaners found it interesting to sit in different location of the class and appreciated the opportunity provided to all learners to be able to sit in the front row of the classroom, where assumed to be the best location for concentration and interaction.

One of the drawback in F-CRS model is that learning heavily relies on technology, which is an internet-based approach and therefore some students faced challenges in joining CRS polling sessions due to tech-related issues or sometimes instable Wi-Fi network connections that is supplied to more than 100 students in the classroom, which subsequently resulted minor dissatisfaction. In addition, few L2 students reported that they found it difficult to work with teammates they did not know in advance and some also highlighted the imbalanced shared responsibilities, which affected the team dynamics. Moreover, few L2 students challenged the suitability of TF-CRS by highlighting the fact that their final written assessment was not based on teamwork. Furthermore, feeling frustrated was expressed by some leaners if unlike most students, they or their team responded to a question incorrectly. The responses also reflected that L1 students’ learning goal is rather exam oriented as they expected IF-CRS model to help them predicting exam questions.

**Figure 3:**
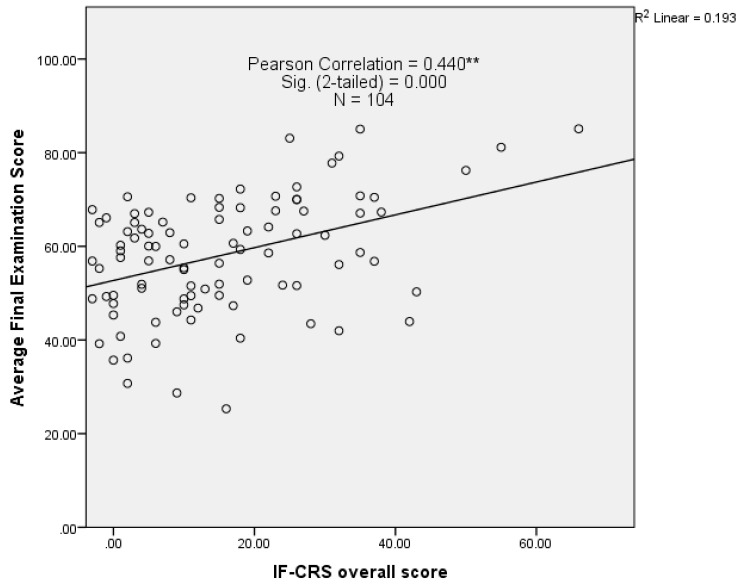
Pearson’s classroom-exam performance correlation in IF-CRS model.

Figure 3 presents correlation analysis between L1 individuals overall score in IF-CRS session and their average final exam score across relevant modules. A significant correlation was found by Pearson-product moment correlation between students’ in class and final examination performance (*r* = 0.440, *p* < 0.01). However, there was not a meaningful exam-classroom performance correlation in TF-CRS model. It is believed that group performance (in-class) affects individual exam scores, in particular when TBL was implemented, to enhance individual learning by creating cognitive friction. Therefore, this contradiction might be due to the misalignment of course learning goals and examination format which requires further investigations in the future studies.

## 4. Discussion

In this study CRS is incorporated as an additional element to our previous blended course design, in which TBL was coupled with FC and demonstrated positive impact on student engagement, course satisfaction and performance [41].

Educators have great concerns that students face significant challenges in adjusting to university life and study after transition from high school, especially if they do not have strong science background [58]. One of the controversial debates in Higher Education is that whether universities acknowledge a role in supporting learners to get used to learning and teaching environments that are different from those at high schools [57]. Barefoot states that Higher Education should be responsible for supporting learners to adapt and help them to respond to the problematic nature of the transition process [59]. Furthermore, students’ engagement with universities has changed due to the technological developments and increased numbers of students who work or are enrolled as part-time students. As a result, supporting university newcomers, keeping them interested and engaged and enhancing their learning experience in new and unfamiliar learning environments are the top aims and priorities for educators [7]. Student engagement is the key factor in learning and academic performance, which is significantly correlated with student in-class concentration, interest, and enjoyment [17,18]. FC approach with balanced asynchronous and synchronous learning environment has supported international students to overcome language barrier and has provided chance for learners to self-study in a preferred place and pace as they can pause, replay, and adjust the speed of the recorded online lectures to their own learning pace [32]. For example, Goh and Ong reported that Malaysian pharmacy students with previous poor academic performance benefited from FC, which proved to improve student pass rate significantly [60]. In addition, CRS provides a technology-enabled learning environment for enhancing interactivity in classroom by collecting, aggregating, and displaying students’ responses [29,31].

Students in this study are all Chinese nationals and mostly had experienced traditional instructor-oriented learning approaches in their past education before starting their higher education in a British college in China that complies with the UK Professional Standards Framework. Therefore, most of them face rocky transition and more challenges than home students when starting their higher education due to cultural and language barriers in learning as well as engagement and interaction difficulties. As such, we hybridised FC model with CRS, in which class performance (individually/ in teams) can be collected and monitored while ongoing feedback were provided during in-class formative assessments [28,29].

As our L1 students were in their first transition stage of becoming active and self-regulated learners, IF-CRS model, which focuses on learning via individual participation and engagement, was introduced without additional complexity that teamwork adds on to the learning environment. Chinese teenagers’ shyness and alienation from family, peers, and school has been reflected in literature and reported by educators [61]. It was hoped that IF-CRS would improve their interactions, overcome engagement barriers, minimise the risk of losing interest and assist junior students to improve their learning while actively but anonymously participate and engage in class activities. TBL model was subsequently blended with FC for second year students, in which CRS was exploited to additionally support peer learning, interactions, and discussion.

Descriptive statistics of student perceptions regarding IF-CRS and TF-CRS approaches show higher satisfaction among L2 group in all elements investigated in the survey in comparison with L1 participants. It is believed that the addition of TBL model to the blended structure of F-CRS model supported L2 students with regards to their academic performance [12,13], peer learning [62], interaction [17], and skills development [18] at a higher extent (Table 2 and Figure 2), which was also in line with student responses in open-ended questions (Table 3). Moreover, L1 negative perceptions might be due to the fact that these learners were new to the UK Higher Education learning and assessment standards and had not fully adapted to their new learning environment.

Time management and self-study skills, as significant contributors to study success, were the skills ranked higher by L1 students, which are frequently reported as issues and challenges for first-year students with regards to course expectations [57]. University students had not encountered the same extent of self-study readings at high school or elsewhere and therefore they need to adopt a more strategic approach to learning tasks, with an emphasis on time management and effective reading strategies, in order to succeed [63]. Self-study and time management skills, as part of “self-regulated learning skills”, are also significantly linked with the flipped part of our teaching model as it requires learners to manage their own time and space so that they are responsible for their own learning prior to attending classes [64]. These skills were also previously reported as the most important improved and/or developed skills by students form Hong Kong studying Information Technology in Education in Cantonese language in a FC model [65]. Moreover, self-study skills, as essential elements in lifelong learning, were reflected in Sun and co-workers’ research who designed a FC model for teaching physics that enhanced students help-seeking ability and supported them to develop their learning vision and goals and to seek help to achieve them [66]. Time management skill can be of extreme value for our students in this study, who are studying a double-degree program, one is taught in Chinese and the other in English.

Problem solving skills were also highly ranked by both groups of students in this study (Figure 2), which are related to CRS activities and formative assessments in IF-CRS and TF-CRS models. Our blended/super BL models also supported our educators to use in-class performance as a diagnostic tool to understand how students responded to the questions and activities and whether the design of the question plays an important role in the development of such skills [67].

In addition, both L1 and L2 students appreciated the incorporation of CRS with regards to their exam preparation and academic performance. Similar findings were observed by Cotner and colleagues teaching biology students. Their study, which investigates the usefulness of CRS in assessment preparation, recommends this system to be incorporated in active learning models due to the ease of use, low cost, effectiveness, and improved classroom climate. Cotner et al. reported that students enjoyed the group interaction and opportunities to learn from each other and appreciated instant feedback on their understanding of course material, which subsequently helped them to improve their academic performance [68]. Similarly, Slain et al. presented the positive impact of CRS in exam performance in clinical pharmacokinetics, medical literature evaluation, pathophysiology and therapeutics modules for pharmacy students [69].

Communication skills were also highly ranked by L2 students who worked in teams in TF-CRS model (Figure 2). This is further supported by strong student perception with regards to the importance of sharing notes and thoughts with peers and its impact on individuals in an interactive and collaborative learning environment (Table 2). This is in line with Ofstad and Brunner study in 2013 that introduced TBL model in a pharmacy course and reported improved communication skills amongst other acquired or developed skills [45].

Open ended questions revealed that our blended/super blended learning and teaching approaches supported students to understand content, prepare for summative assessment and promote their engagement and interaction. However, respondents also highlighted the technical issues related to connectivity to CRS online platform and the limited time allocated to respond to each question (Table 3). Although specific sessions provided to answer logistical queries about CRS sessions, further investigation around learners’ internet supplier (phone/University WiFi), the type of their smart device and their internet browser as well as issues related to the registration process would be beneficial. We tended to limit the time answering questions as CRS questions were supposed to simulate exam questions and enhance constructive competition between individuals and teams. In addition, although the majority of learners perceived IF-CRS and TF-CRS models as suitable approaches to develop and enhance their skills and improve their learning, some L1 and L2 students stated that they would prefer working in teams and individually, respectively. Those L1 students reported that working in teams can improve their communication skills, which indicates that they welcome collective activities and peer interaction in their learning and development. L2 students, who preferred to work individually, faced challenges working in team as a result of poor team dynamics and lack of adequate and shared responsibilities of their teammates. Few L2 respondents also indicated that they would prefer to focus on CRS activities individually rather being involved in group tasks as exam is an individual measure (Table 3). This shows that those students in this study were rather exam-orientated, which might be due to the local and academic cultures students operate in within China, involving mostly teacher-centered primary and secondary education, which students have experienced up to this point of their academic careers [43]. All these variables play a significant part in student development, which are correlated with Level 1 (Table 2 and Table 3) and Level 2 (Figure 2) Kirkpatrick’s models of course evaluation that measures students’ reactions to the program (Level 1) and their development in skills, knowledge, and attitude (Level 2) [70]. We hope that students gradually understand the potential advantages of working in teams as they may not have had enough experience of team-work and may not be fully aware of its long-term benefit in which great ideas are shared, course enjoyment is enhanced, and communication skills are improved.

As CRS offers the ability to track student academic progress throughout the year, it was essential to investigate the correlation between student classroom and exam performance. Our main focus was primarily on L1 performance correlation as we found that teams classroom performance is significantly higher than even the students, on the teams, whose individual performances on the same assessments were the highest of all team members. This is consistent with a growing literature indicating that teams outperform every member of the teams on assessment [71,72]. As a result, a transparent correlation of L2 student classroom-exam performance was not justified. The Pearson correlation coefficient [73] indicated that L1 students who scored higher in class, also achieved higher examination results. Moreover, scatter points revealed students with high exam performance and low classroom score, which might be an evidence that the use of immediate classroom feedback via CRS had a positive impact on final examination performance [74].

## 5. Conclusions

Our teaching models provide flexible learning frameworks that guide to address learners’ variability. Sustained engagement achieved by proving frequent feedback and building a community through multiple opportunities to communicate with educators and peers. F-CRS model created autonomy for individuals and teams how to make choices while also gave more responsibility to learners for planning, direction, demonstration, and advocacy of learning.

It is understood that evaluation of the course formats is restricted to student perception and therefore it appears to be essential to study short- and long-term learning effects and transfer of knowledge in the future in order to decide whether the investment in these complex course designs is warranted. Further investigations will also focus on greater utilisation of the F-CRS model within the curricula, such as the implementation of F-CRS in practical component of the course, or enabling learners to allocate and form their teams in order to overcome the barriers they have faced regarding the team dynamics and sharing responsibilities in randomly allocated teams in TF-CRS model. It is also worth identifying what parts of the course within the curriculum require IF-CRS, and what parts are better to be instructed via TF-CRS as some tasks may not need to be completed in teams and may have counterproductive effects on both individual learners, the team and even on organisation. Finally, it is recommended that any new learning strategy to be implemented from the beginning of the course and continued throughout the learners’ studies, helping the learners to adapt to the new educational strategies and allowing for enough time to deal with challenges and identify limitations.

## Figures and Tables

**Table 1:** Demographic information of IF-CRS and TF-CRS participants.

Demographic Variable		IF-CRS	TF-CRS
Gender	Male	25%	18%
	Female	75%	82%
Age	17–19	42%	12%
	20–22	58%	88%
Enrolled degree programme	BSc Pharmaceutical Biotechnology	50%	36%
	BSc Pharmaceutical Sciences	50%	64%

**Table 2:** Descriptive statistics of student perception regarding IF-CRS and TF-CRS approaches.

Questions	IF-CRS	TF-CRS
	Mean ± SD	
**Student Performance**		
This method of teaching is a useful way to study	4.11 ± 0.78	4.52 ± 0.80
IF-CRS/TF-CRS helped me to remember information well	3.97 ± 0.91	4.39 ± 0.82
Receiving scores after sessions helped me to work on my progression	3.97 ± 0.88	4.00 ± 1.03
**Assessment**		
This method allows me to perform better in my examinations than if I had been taught in traditional lectures	3.72 ± 1.11	4.21 ± 0.90
Imposing negative marking for MCQs in F-CRS sessions encouraged me to answer the questions that I am quite confident about	3.54 ± 0.08	3.95 ± 0.94
Imposing negative marking for MCQs in F-CRS sessions helped me to practice and understand the format of final exam questions	3.67 ± 1.15	3.84 ± 0.97
**F-CRS Comparison with Traditional Teaching**		
Being taught in this way encouraged me to ask questions in the class	3.81 ± 1.01	4.18 ± 0.77
Graded F-CRS activities motivated me to engage in class more actively	4.03 ± 0.77	4.24 ± 0.90
I prefer IF-CRS/TF-CRS teaching to traditional lectures	3.67 ± 1.07	4.21 ± 0.99
IF-CRS/TF-CRS learning has increased my interest in the course	3.89 ± 0.75	4.18 ± 0.98
This model encouraged me to attend classes more than traditional lectures	4.03 ± 0.70	4.27 ± 0.88
**Usefulness of F-CRS Sessions**		
Preparation before sessions helped me to understand information more in the class than if I had just heard about it for the first time during the class	3.92 ± 0.97	4.33 ± 0.60
CRS sessions helped me to understand the information more fully I had prepared in advance	3.92 ± 0.91	4.36 ± 0.60
The frequency of responses shown on screen after pooling was closed motivated me to be involved within the F-CRS activities	3.92 ± 0.77	4.12 ± 0.86
**Peers Learning, Interaction, and Development**		
F-CRS sessions made me want to learn from my peers	3.97 ± 0.81	4.36 ± 0.70
F-CRS sessions allowed me to learn from my peers	3.92 ± 0.87	4.27 ± 0.67
I am happy to share class notes and appropriate study materials with my peers during F-CRS exercises	3.94 ± 0.86	4.45 ± 0.62
I believe that the feedback I provided to my peers during F-CRS sessions will assist with their professional development	3.81 ± 0.86	4.42 ± 0.56
I believe that the feedback I provided to my peers during F-CRS sessions will assist with their academic development	3.83 ± 0.81	4.27 ± 0.58
**Leaving Comfort Zone and Engage**		
F-CRS competition motivated me to be more active within the sessions	3.86 ± 0.90	4.03 ± 1.01
Answering questions anonymously during F-CRS sessions motivated me to engage more within the activities	4.06 ± 0.63	4.33 ± 0.78
**Recommended Teaching Approach**		
There should be more IF-CRS/TF-CRS learning included within my course	3.75 ± 1.05	4.21 ± 0.857

**Table 3:** Examples of students’ positive and negative comments towards the use of IF-CRS and TF-CRS models.

Question	IF-CRS	TF-CRS
What did you like about IF-CRS/TF-CRS learning model?	▪ *“I think IF-CRS is great. It can help teachers to know if we completely understand the course and familiarises us with the format of exam questions.”* ▪ *“It is a way to force me to preview the lectures before the class and it can help me to understand more about the content of the lectures.”* ▪ *“It is a good way to exercise before and during lectures.”*	▪ *“I like this new teaching approach. It is very helpful for us to master the key points and review the knowledge in time.”* ▪ *“It can help me keep focus on the class and preview before class with my team.”* ▪ *“I enjoyed the team works as well as group discussion. And sitting on different seats also promoted my learning.”* ▪ *“It provided a question bank that we can practice for the exam.”*
What did you not like about IF-CRS/ TF-CRS learning model?	▪ *“Some questions are difficult for me to understand.”* ▪ *“I cannot always connect to TurningPoint.”* ▪ *“Only a small number of the practiced questions appear in the real exam.”*	▪ *“Sometimes the WiFi is not very stable to connect TurningPoint App.”* ▪ *“Grouping is mandatory.”* ▪ *“I don’t want to cooperate with people I’m not familiar with or I don’t like, which affects my mood in the class.”*
What did you like about CRS activities in general?	▪ *“I could see my score instantly.”* ▪ *“It increased my interest to join the class activities.”* ▪ *“It is like a class summary, which helps to systematise the main points.”*	▪ *“It encouraged us to show our thoughts to other people.”* ▪ *“I can get some question bank that It is beneficial to my study.”* ▪ *“Combining the questions with the coursework improved my understanding about the course.”*
What did you not like about CRS activities in general?	▪ *“It is sometimes difficult to operate.”* ▪ *“I was afraid of being the only one or within few students who make mistakes.”*	▪ *“Difficult to login.”* ▪ *“I felt uneasy when our team answered the question incorrectly.”*

## Abbreviations

ARS	Audience Response System
BL	Blended learning
CQC	China Medical University-The Queen’s University Belfast joint College
CRS	Classroom Response system
FC	Flipped Classroom
F-CRS	Flipped classroom coupled with classroom response system
IF-CRS	F-CRS model implemented for individuals
iRAT	Readiness Assessment Test for Individuals
L1	Level one BSc students
L2	Level two BSc students
MPharm	Master of Pharmacy
PharmD	Doctor of Pharmacy
PRS	Personal Response System
QUB	Queen’s University Belfast
TBL	Team-Based Learning
TF-CRS	F-CRS model implemented for Teams
TP	TurningPoint classroom response system technology
tRAT	Readiness Assessment Test for teams
